# Association Between Depressive Symptoms and Incidence of Stroke in a Population With Cardiovascular-Kidney-Metabolic Syndrome Stages 0 to 3: Nationwide Prospective Cohort Study

**DOI:** 10.2196/80988

**Published:** 2026-04-01

**Authors:** Yunjie Li, Lin Wang, Lujing Gao

**Affiliations:** 1 Department of Radiology, Zhuzhou Clinical College, Jishou University, Zhuzhou, China; 2 Department of Respiratory and Critical Care Medicine, Zhuzhou Hospital Affiliated to Xiangya School of Medicine, Central South University, Zhuzhou, China; 3 Department of Radiology, Zhuzhou Hospital Affiliated to Xiangya School of Medicine, Central South University, Zhuzhou, China, 86 15171791199

**Keywords:** stroke, cardiovascular-kidney-metabolic syndrome, CKM, depression, restricted cubic spline analysis, RCS, subgroup analysis

## Abstract

**Background:**

The association between depressive symptoms and cardiovascular diseases is well established. However, their impact on the incidence of stroke in individuals with cardiovascular-kidney-metabolic (CKM) syndrome remains unclear.

**Objective:**

This study aims to investigate the impact of depressive symptoms at different stages of CKM syndrome on the incidence of new-onset stroke.

**Methods:**

This study used data from the China Health and Retirement Longitudinal Study. Depressive symptoms at baseline were assessed using the Center for Epidemiologic Studies Depression Scale, with stroke incidence determined through standardized follow-up questionnaires. Cox regression and restricted cubic spline regression were used to evaluate the association between depressive symptoms and stroke risk.

**Results:**

The analysis included 9593 participants (n=5180, 54.92% male; mean age of 60.89, SD 9.39 y), classified into CKM stages 0 to 3. Fully adjusted Cox regression showed that each 1-point increase in depressive score was associated with a 3% higher stroke risk (hazard ratio 1.03, 95% CI 1.02‐1.04; *P*<.001). Restricted cubic spline regression confirmed a significant positive linear relationship between depressive symptoms and stroke incidence (*P*<.001).

**Conclusions:**

This cohort study demonstrates a positive linear association between depressive symptoms and increased stroke incidence in individuals with CKM syndrome (stages 0‐3). These findings highlight the importance of emotional health management, suggesting that effective depression treatment may help reduce stroke risk through inflammation reduction and lifestyle improvements.

## Introduction

In the October 2023 Presidential Advisory, the American Heart Association (AHA) defined cardiovascular-kidney-metabolic (CKM) syndrome as a systemic condition characterized by complex pathophysiological interactions among metabolic risk factors (eg, obesity and diabetes), chronic kidney disease (CKD), and cardiovascular disease (CVD). These interrelated processes contribute to multiorgan dysfunction and significantly increase the risk of adverse cardiovascular events. Investigating CKM syndrome as an integrated condition offers a strong scientific foundation for developing comprehensive diagnostic and therapeutic strategies aimed at improving patient outcomes [1].

Metabolic disorders, by reducing insulin sensitivity, elevate the risk of dyslipidemia in patients with CKM, often leading to early cardiovascular complications such as coronary atherosclerosis [2,3], myocardial infarction [4], and heart failure [5]. Additionally, the interaction between CKD and CVD can trigger inflammatory responses [6], oxidative stress [7], and endothelial dysfunction [8]. Moreover, extensive evidence indicates that CVD constitutes the primary clinical burden associated with CKM syndrome, with a disproportionately high impact [9]. Compared to healthy individuals, patients with CKM experience more complex pathophysiological changes.

Conventional medical practice often diagnoses and treats CVD, CKD, and metabolic disorders separately, overlooking their interrelationships. AHA emphasizes the importance of early detection of CKM staging in the preclinical phase [1]. Previous studies have shown that adults in the United States have concurrent cardiovascular, kidney, and metabolic diseases, with this prevalence on the rise [10]. Roth et al [11] observed that in patients undergoing noncardiac surgery, the risk of postoperative adverse outcomes increases with higher CKM staging. Certain pharmacological interventions, such as angiotensin-converting enzyme inhibitors or angiotensin II receptor blockers, sodium-glucose co-transporter 2 inhibitors, and nonsteroidal mineralocorticoid receptor antagonists, not only slow the progression of CKD but also reduce mortality from CVD [12]. These findings suggest that the 2 conditions may share common biological pathways. A growing body of evidence suggests that the disproportionate clinical burden of CKM syndrome is strongly linked to CVD [1,13]. Stroke is the second-leading cause of death and the third-leading cause of disability globally [14]. Additionally, it is estimated that 2 million new cases of stroke occur annually in China, with 70% to 80% of affected individuals experiencing disabilities that render them incapable of independent living [15]. Studying the incidence of stroke in populations at different stages of CKM syndrome holds significant clinical and public health value.

In 2013, depression accounted for more than 10 million disability-adjusted life years in China, which is projected to rise by approximately 10% by 2025 [16]. Negative emotional states, such as anxiety, depression, or chronic stress, trigger hyperactivation of the hypothalamic-pituitary-adrenal axis, resulting in elevated cortisol levels that exacerbate metabolic imbalances [17]. This physiological disturbance can lead to increased blood pressure, endothelial dysfunction, and the development of atherosclerosis [18]. Moreover, individuals with depression often exhibit systemic low-grade inflammation, which impairs insulin signaling and contributes to dysregulated glucose metabolism [19]. This condition further promotes vascular endothelial inflammation and oxidative stress, accelerating vascular stiffness and plaque formation [20]. Previous studies have shown a strong association between depression and elevated proinflammatory cytokines (eg, interleukin-6 and C-reactive protein [CRP]) in patients with CKD, potentially increasing the risk of cardiovascular events and overall mortality [21]. A 2-year observational study reported that persistent depressive symptoms elevate the risk of stroke, while improvements in these symptoms reduce its incidence [22]. However, the relationship between depressive symptoms and stroke incidence in the population with CKM remains inadequately explored.

Clarifying the association between depressive symptoms and stroke incidence in this group could inform targeted psychological interventions and disease management strategies, ultimately improving patient outcomes and overall health. This study aims to investigate the impact of depressive symptoms at different stages of CKM syndrome on the incidence of new-onset stroke. Since clinical criteria for CVD are present in CKM stage 4, we selected individuals in CKM stages 0 to 3 as the target population for this study.

## Methods

### Ethical Considerations

The studies involving human participants adhered to the principles of the Declaration of Helsinki and were reviewed and approved by the ethics review committee of Peking University (IRB00001052-11015). The participants provided their written informed consent to participate in this study. To ensure privacy and confidentiality, all data accessed from the China Health and Retirement Longitudinal Study (CHARLS) database were deidentified and analyzed anonymously.

### Data Source and Study Population

The data for this study were obtained from the CHARLS, a national cohort study covering urban and rural areas across 28 provinces in China. CHARLS is representative of the population aged 45 years and older in China. All participants provided written informed consent before participating in the study. The baseline survey for CHARLS began in 2011, including approximately 17,000 respondents from over 10,000 households. A stratified sampling method was used to ensure national representativeness. Follow-up surveys were conducted in 2013, 2015, and 2018. All field staff underwent systematic and professional training and conducted face-to-face interviews using standardized questionnaires. The CHARLS database includes information across 5 key domains: demographic and household characteristics, health status, economic status, health care use and expenditures, and social participation and psychological well-being. In this study, the baseline was set in 2011, with the follow-up period ending in 2018.

### Exposure

The Center for Epidemiologic Studies Depression Scale is a widely used self-report instrument designed to assess depressive symptoms in the general population [23]. The scale contains 10 items measuring depressive symptoms experienced over the past week, including mood states, somatic symptoms, interpersonal difficulties, and positive affect (reverse-scored). Each item is rated on a 4-point Likert scale: 0 (very little or none of the time), 1 (not much; 1‐2 d), 2 (sometimes or about half the time; 3‐4 d), and 3 (most of the time; 5‐7 d). The total score ranges from 0 to 30, with higher scores indicating more severe depressive symptoms. Participants were categorized into four groups based on quartiles of depression scores: the first group ranged from 0 to 3, the second group from 3 to 7, the third group from 7 to 12, and the fourth group from 12 to 30.

### Outcome

The primary outcome of our study was the incidence of new-onset stroke events. A confirmed diagnosis of stroke during the follow-up period was classified as the first event, with the reported date recorded as the onset date. The timing of the stroke was determined by calculating the time interval from the baseline assessment to the reported onset date. For participants who did not report a stroke, the follow-up time was calculated as the interval between the baseline assessment and the date of their last survey. Alternatively, if data loss occurred during this period, the year in which the loss was first detected was considered the follow-up year.

### Definition of CKM Syndrome Stages 0 to 3

The classification of CKM syndrome stages 0 to 3 is based on the guidelines outlined in the AHA Presidential Advisory Statement on CKM Syndrome [1], while also referencing the CKM classification criteria from the study by Zheng et al [24].

Stage 0 is defined as individuals with no CKM risk factors, characterized by normal BMI, waist circumference, blood glucose levels, blood pressure, and lipid levels, as well as the absence of CKD and CVD.

Stage 1 includes individuals with an elevated BMI (≥23 kg/m² for Asian populations) and an elevated waist circumference (≥80 cm for women; ≥90 cm for men), or prediabetes (defined as a glycated hemoglobin of 5.7% to <6.5% or a fasting blood glucose of 100 mg/dL to <126 mg/dL) without the presence of CKD.

Stage 2 is defined as individuals with metabolic risk factors (eg, hypertriglyceridemia [≥135 mg/dL], hypertension, metabolic syndrome [MetS]) or CKD of moderate-to-high risk and diabetes. MetS is defined by the presence of three or more of the following: (1) ≥80 cm for women and ≥90 cm for men; (2) high-density lipoprotein (HDL) <40 mg/dL for men and <50 mg/dL for women; (3) triglycerides ≥150 mg/dL; (4) elevated blood pressure (systolic blood pressure ≥130 mmHg or diastolic blood pressure ≥80 mmHg and/or use of antihypertensive medications); and (5) fasting blood glucose ≥100 mg/dL.

Stage 3 is characterized by individuals with very-high-risk CKD stages or subclinical atherosclerotic CVD with a predicted 10-year CVD risk of less than 10%. Following the methodology of Deng et al [24], atherosclerotic CVD risk was determined using the Framingham risk score. The estimated glomerular filtration rate (eGFR) was calculated via the Chinese Modification of Diet in Renal Disease equation [25].

### Covariates

We collected data on various independent variables, including demographic characteristics such as age, gender, educational level, BMI, smoking status, alcohol consumption, and place of residence. Blood test parameters such as CRP, uric acid (UA), blood urea nitrogen (BUN), glucose, hemoglobin A_1c_ (HbA_1c_), HDL, non-HDL, low-density lipoprotein (LDL), total cholesterol (TC), triglycerides, eGFR, and platelet count were collected. Additionally, we gathered information on disease conditions, including hypertension, diabetes, CVD, chronic liver disease, sleep disorders, lung disease, and kidney disease. Sleep quality was assessed using the question, “My sleep in the last week was restless,” with 3 response options: little or no time (<1 d), some of the time (1‐2 d), and occasional or moderate time (3‐4 d). Participants who answered ≥1 were identified as having a sleep problem. The selection of covariates in this study was based on established theoretical frameworks and prior epidemiological evidence regarding risk factors for both depression [26,27] and CVDs [24].

### Statistical Analysis

Participants in this study were categorized into 4 quartiles (Q1-Q4) based on baseline depressive scores: Q1 (0‐3), Q2 (4-7), Q3 (8-12), and Q4 (13-30). Continuous variables with a normal distribution were presented as mean (SD), while categorical variables were expressed as frequencies and percentages. Differences between groups were evaluated using the chi-square test and the independent 2-sample 2-tailed *t* test for continuous variables. To explore the association between depressive symptoms and stroke across various demographic characteristics, subgroup analyses were performed for categorical variables such as education level, gender, smoking status, drinking status, and CKM syndrome stages (stages 0-3). The univariate and multivariate Cox regression models were applied to estimate hazard ratio (HR) and 95% CIs for stroke risk associated with baseline depressive scores. Additionally, restricted cubic spline (RCS) regression was used to assess potential nonlinear relationships between depressive scores and stroke incidence in the overall population with CKM syndrome (stages 0‐3) and specifically within CKM stage 2 and stage 3 subgroups. All statistical analyses were performed using R software (version 4.4.0; R Foundation for Statistical Computing), with a 2-sided *P* value<.05 considered statistically significant.

## Results

### Baseline Characteristics of Participants

**Figure 1.**
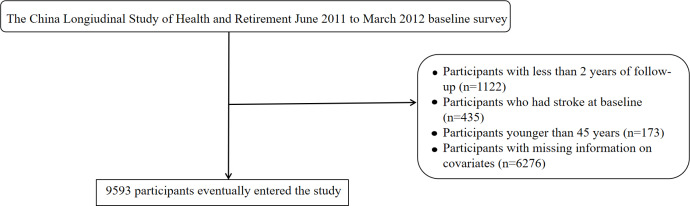
Flowchart illustrating the inclusion and exclusion criteria for the study population.

In the 2011 baseline survey, 17,705 participants were initially included. After excluding individuals with missing stroke data, 17,596 participants remained. Further exclusions were made for those with less than 2 years of follow-up (n=1122) and those with a history of stroke at baseline (n=435), leaving a total of 16,039 participants, of whom 12,852 were classified in CKD stages 0 to 3. Subsequently, 173 participants under the age of 45 years were removed. After removing individuals with missing covariate data, 9593 participants were retained for the final analysis (Figure 1).

Among them, 54.92% (n=5268) were male individuals. Baseline characteristics, as shown in Table 1, are presented according to the quartiles of the depressive score. The analysis revealed that women, married individuals, and those with lower educational attainment were more susceptible to depression compared to men, unmarried individuals, and those with higher levels of education. Specifically, there was a stark contrast in the distribution of education levels across depression quartiles: individuals with primary school education or below accounted for 82.18% of the most severe depressive group (Q4), whereas those with a college degree or higher represented only 0.95% of the same group. As depressive symptoms increased, the proportion of women also increased, while the proportion of men decreased (*χ*²_3_=186.7; *P*<.001). Similarly, the proportion of individuals with lower educational attainment rose, while the proportion of those with higher education decreased (*χ*²_3_=186.70; *P*<.001), with these differences being statistically significant. Furthermore, individuals with chronic conditions such as CVD, liver disease, kidney disease, and lung disease were more likely to experience depressive symptoms than those without these conditions. Additionally, these individuals were more frequently represented among those with the most severe depressive symptoms (Q4). Specifically, individuals with newly diagnosed stroke exhibited significantly more severe depressive symptoms (mean 9.49, SD 6.53) at the 2011 baseline compared to those without stroke (mean 8.16, SD 6.17). Patients with newly diagnosed stroke had significantly higher levels of age, BMI, LDL, HDL, glucose, triglycerides, TC, UA, CRP, and HbA_1c_ compared to healthy controls. In contrast, their sleep duration and eGFR were significantly lower in patients newly diagnosed with stroke compared to healthy controls (Table 2).

**Table 1.** Baseline characteristics of individuals classified by quartiles of the depressive score (N=9593)^a^.

Variable	Total (N=9593)	Q1 (n=2495)	Q2 (n=2572)	Q3 (n=2304)	Q4 (n=2222)	*F* test or chi-square (*df*)	*P* value
Age (y), mean (SD)	60.89 (9.39)	59.63 (9.09)	60.72 (9.55)	61.22 (9.57)	62.17 (9.15)	30.34 (3)^b^	<.001
Sex, n (%)						186.70 (3)^c^	<.001
Female	4325 (45.08)	916 (36.71)	1072 (41.68)	1105 (47.96)	1232 (55.45)		
Male	5268 (54.92)	1579 (63.29)	1500 (58.32)	1199 (52.04)	990 (44.55)		
Marital status, n (%)						115.47 (3)^c^	<.001
Married	8433 (87.91)	2282 (91.46)	2314 (89.97)	2017 (87.54)	1820 (81.91)		
Unmarried	1160 (12.09)	213 (8.54)	258 (10.03)	287 (12.46)	402 (18.09)		
Education, n (%)						350.22 (9)^c^	<.001
College school and higher	335 (3.49)	154 (6.17)	92 (3.58)	68 (2.95)	21 (0.95)		
High school	598 (6.23)	227 (9.10)	192 (7.47)	108 (4.69)	71 (3.20)		
Middle school	1899 (19.80)	619 (24.81)	562 (21.85)	414 (17.97)	304 (13.68)		
Primary school or below	6761 (70.48)	1495 (59.92)	1726 (67.11)	1714 (74.39)	1826 (82.18)		
Location, n (%)						151.49 (3)^c^	<.001
Rural	6293 (65.60)	1446 (57.96)	1632 (63.45)	1561 (67.75)	1654 (74.44)		
Urban	3300 (34.40)	1049 (42.04)	940 (36.55)	743 (32.25)	568 (25.56)		
BMI (kg/m²), mean (SD)	23.69 (3.93)	24.08 (3.88)	23.90 (3.93)	23.44 (3.84)	23.25 (4.03)	22.95 (3)^b^	<.001
Waist, mean (SD)	85.24 (12.80)	86.01 (12.95)	85.83 (12.94)	84.66 (12.50)	84.31 (12.67)	10.35 3)^b^	<.001
Smoking status, n (%)						52.65 (6)^c^	<.001
Former smoke	982 (10.24)	240 (9.62)	274 (10.65)	249(10.81)	219 (9.86)		
Never smoke	5231 (54.53)	1249 (50.06)	1382 (53.73)	1288 (55.90)	1312 (59.05)		
Smoke	3380 (35.23)	1006 (40.32)	916 (35.61)	767 (33.29)	691 (31.10)		
Drinking status, n (%)						91.61 (3)^c^	<.001
Drink	3448 (35.94)	1033 (41.40)	979 (38.06)	802 (34.81)	634 (28.53)		
No drink	6145 (64.06)	1462 (58.60)	1593 (61.94)	1502 (65.19)	1588 (71.47)		
Sleep problem, n (%)						6.89 (3)^c^	.08
No	13 (0.14)	2 (0.08)	2 (0.08)	2 (0.09)	7 (0.32)		
Yes	9580 (99.86)	2493 (99.92)	2570 (99.92)	2302 (99.91)	2215 (99.68)		
Platelets, mean (SD)	210.88 (75.38)	206.05 (64.24)	210.10 (71.52)	212.59 (86.52)	215.45 (78.57)	6.63 (3)^b^	<.001
CRP^d^, mean (SD)	2.69 (6.82)	2.65 (7.07)	2.60 (5.95)	2.97 (8.58)	2.53 (5.23)	1.92 (3)^b^	.12
Glucose, mean (SD)	112.64 (39.48)	113.86 (40.61)	111.90 (36.90)	112.22 (39.67)	112.59 (40.85)	1.18 (3)^b^	.31
HbA_1c_^e^, mean (SD)	5.32 (0.87)	5.30 (0.87)	5.33 (0.85)	5.31 (0.81)	5.35 (0.97)	1.61 (3)^b^	.19
BUN^f^, mean (SD)	15.89 (4.53)	15.84 (4.35)	15.97 (4.55)	15.96 (4.70)	15.79 (4.53)	0.88 (3)^b^	.45
UA^g^, mean (SD)	4.56 (1.27)	4.70 (1.28)	4.64 (1.29)	4.53 (1.27)	4.36 (1.19)	32.5 (3)^b^	<.001
TC^h^, mean (SD)	195.73 (38.44)	195.65 (37.77)	195.62 (38.94)	194.86 (39.26)	196.87 (37.73)	1.06 (3)^b^	.37
HDL^i^, mean (SD)	50.59 (15.27)	49.49 (14.97)	50.28 (15.05)	50.88 (15.20)	51.87 (15.82)	10.16 (3)^b^	<.001
LDL^j^, mean (SD)	117.11 (35.88)	117.27 (35.87)	116.98 (35.83)	116.39 (36.49)	117.80 (35.33)	0.61 (3)^b^	.61
Triglycerides, mean (SD)	141.23 (104.29)	145.10 (106.06)	141.91 (107.22)	139.07 (98.79)	138.34 (104.32)	2.08 (3)^b^	.10
Non-HDL, mean (SD)	145.15 (39.02)	146.16 (38.48)	145.34 (39.98)	143.98 (39.32)	145.00 (38.15)	1.28 (3)^b^	.28
eGFR^k^, mean (SD)	120.08 (29.53)	120.73 (29.22)	119.13 (26.83)	119.93 (32.70)	120.61 (29.36)	1.56 (3)^b^	.20
Hypertension, n (%)						27.73 (3)^c^	<.001
No	6447 (67.21)	1764 (70.70)	1744 (67.81)	1523 (66.10)	1416 (63.73)		
Yes	3146 (32.79)	731 (29.30)	828 (32.19)	781 (33.90)	806 (36.27)		
Diabetes, n (%)						26.37 (3)^c^	<.001
No	8860 (92.36)	2351 (94.23)	2392 (93.00)	2097 (91.02)	2020 (90.91)		
Yes	733 (7.64)	144 (5.77)	180 (7.00)	207 (8.98)	202 (9.09)		
Lung disease, n (%)						129.58 (3)^c^	<.001
No	8576 (89.40)	2343 (93.91)	2325 (90.40)	2045 (88.76)	1863 (83.84)		
Yes	1017 (10.60)	152 (6.09)	247 (9.60)	259 (11.24)	359 (16.16)		
Liver disease, n (%)						20.10 (3)^c^	<.001
No	9296 (96.90)	2444 (97.96)	2497 (97.08)	2228 (96.70)	2127 (95.72)		
Yes	297 (3.10)	51 (2.04)	75 (2.92)	76 (3.30)	95 (4.28)		
CVD^l^, n (%)						95.58 (3)^c^	<.001
No	8518 (88.79)	2309 (92.55)	2326 (90.44)	2014 (87.41)	1869 (84.11)		
Yes	1075 (11.21)	186 (7.45)	246 (9.56)	290 (12.59)	353 (15.89)		
Kidney disease, n (%)						44.41 (3)^c^	<.001
No	9188 (95.78)	2418 (96.91)	2486 (96.66)	2209 (95.88)	2075 (93.38)		
Yes	405 (4.22)	77 (3.09)	86 (3.34)	95 (4.12)	147 (6.62)		
CKM^m^ stage, n (%)						14.63 (9)^c^	.10
0	985 (10.27)	248 (9.94)	255 (9.91)	255 (11.07)	227(10.22)		
1	1817 (18.94)	502 (20.12)	523 (20.33)	407 (17.66)	385 (17.33)		
2	3999 (41.69)	1023 (41.00)	1038 (40.36)	980 (42.53)	958 (43.11)		
3	2792 (29.10)	722 (28.94)	756 (29.39)	662 (28.73)	652 (29.34)		

a Data are presented as the mean (SD), median (IQR), or number (%), as appropriate.

b *F* values from 1-way ANOVA.

c Chi-square test.

d CRP: C-reactive protein.

e HbA_1c_: hemoglobin A_1c_.

f BUN: blood urea nitrogen.

g UA: uric acid.

h TC: total cholesterol.

i HDL: high-density lipoprotein.

j LDL: low-density lipoprotein.

k eGFR: estimated glomerular filtration rate.

l CVD: cardiovascular disease.

m CKM: cardiovascular-kidney-metabolic.

**Table 2.** Baseline characteristics of individuals classified by onset of stroke (N=9593).

Variable	Total (N=9593)	No (n=8549)	Yes (n=1044)	Chi-square or *t* test (*df*)	*P* value
Age (y), mean (SD)	60.89 (9.39)	60.73 (9.48)	62.25 (8.46)	−5.43 (9591)^a^	<.001
Sex, n (%)				0.64 (1)^b^	.42
Female	4325 (45.08)	3867 (45.23)	458 (43.87)		
Male	5268 (54.92)	4682 (54.77)	586 (56.13)		
Marital status, n (%)				0.87 (1)^b^	.35
Married	8433 (87.91)	7525 (88.02)	908 (86.97)		
Unmarried	1160 (12.09)	1024 (11.98)	136 (13.03)		
Education, n (%)				7.04 (3)^b^	.07
College school and higher	335 (3.49)	295 (3.45)	40 (3.83)		
High school	598 (6.23)	533 (6.23)	65 (6.23)		
Middle school	1899 (19.80)	1724 (20.17)	175 (16.76)		
Primary school or below	6761 (70.48)	5997 (70.15)	764 (73.18)		
Location, n (%)				0.05 (1)^b^	.82
Rural	6293 (65.60)	5612 (65.65)	681 (65.23)		
Urban	3300 (34.40)	2937 (34.35)	363 (34.77)		
BMI, mean (SD)	23.69 (3.93)	23.57 (3.91)	24.66 (3.98)	−8.37 (9591)^a^	<.001
Waist, mean (SD)	85.24 (12.80)	84.86 (12.71)	88.41 (13.04)	−8.32 (9591)^a^	<.001
Smoking status, n (%)				6.12 (2)^b^	.05
Former smoke	982 (10.24)	853 (9.98)	129 (12.36)		
Never smoke	5231 (54.53)	4684 (54.79)	547 (52.39)		
Smoke	3380 (35.23)	3012 (35.23)	368 (35.25)		
Drinking status, n (%)				2.86 (1)^b^	.09
Drink	3448 (35.94)	3098 (36.24)	350 (33.52)		
No drink	6145 (64.06)	5451 (63.76)	694 (66.48)		
Sleep problem, n (%)				0.66 (1)^b^	.41
No	13 (0.14)	13 (0.15)	0（0）	
Yes	9580 (99.86)	8536 (99.85)	1044 (100.00)		
Platelets, mean (SD)	210.88 (75.38)	209.54 (71.86)	221.90 (99.04)	−3.91 (9591)^a^	<.001
CRP^c^, mean (SD)	2.69 (6.82)	2.59 (6.49)	3.44 (9.10)	−2.91 (9591)^a^	.008
Glucose, mean (SD)	112.64 (39.48)	111.92 (38.29)	118.58 (47.72)	−4.34 (9591)^a^	<.001
HbA_1c_, mean (SD)	5.32 (0.87)	5.30 (0.86)	5.45 (0.97)	−4.66 (9591)^a^	<.001
BUN^e^, mean (SD)	15.89 (4.53)	15.89 (4.54)	15.90 (4.45)	−0.08 (9591)^a^	.94
UA^f^, mean (SD)	4.56 (1.27)	4.55 (1.26)	4.70 (1.34)	−3.45 (9591)^a^	<.001
TC^g^, mean (SD)	195.73 (38.44)	195.32 (38.54)	199.11 (37.43)	−3.08 (9591)^a^	.006
HDL^h^, mean (SD)	50.59 (15.27)	50.88 (15.26)	48.16 (15.16)	5.46 (9591)^a^	<.001
LDL^i^, mean (SD)	117.11 (35.88)	116.80 (35.83)	119.59 (36.26)	−2.35 (9591)^a^	.02
Triglycerides, mean (SD)	141.23 (104.29)	139.35 (102.50)	156.66 (116.83)	−4.58 (9591)^a^	<.001
Non-HDL, mean (SD)	145.15 (39.02)	144.44 (39.12)	150.95 (37.69)	−5.25 (9591)^a^	<.001
eGFR^j^, mean (SD)	120.08 (29.53)	120.63 (29.79)	115.57 (26.89)	5.67 (9591)^a^	<.001
Depressive score, mean (SD)	8.31 (6.23)	8.16 (6.17)	9.49 (6.53)	−6.24 (9591)^a^	<.001
Hypertension, n (%)				232.02 (1)^b^	<.001
No	6447 (67.21)	5964 (69.76)	483 (46.26)		
Yes	3146 (32.79)	2585 (30.24)	561 (53.74)		
Diabetes, n (%)				54.33 (1)^b^	<.001
No	8860 (92.36)	7956 (93.06)	904 (86.59)		
Yes	733 (7.64)	593 (6.94)	140 (13.41)		
Lung disease, n (%)				0.20 (1)^b^	.66
No	8576 (89.40)	7638 (89.34)	938 (89.85)		
Yes	1017 (10.60)	911 (10.66)	106 (10.15)		
Liver disease, n (%)				4.48 (1)^b^	.03
No	9296 (96.90)	8296 (97.04)	1000 (95.79)		
Yes	297 (3.10)	253 (2.96)	44 (4.21)		
CVD^k^, n (%)				70.01 (1)^b^	<.001
No	8518 (88.79)	7672 (89.74)	846 (81.03)		
Yes	1075 (11.21)	877 (10.26)	198 (18.97)		
Kidney disease, n (%)				17.18 (1)^b^	<.001
No	9188 (95.78)	8214 (96.08)	974 (93.30)		
Yes	405 (4.22)	335 (3.92)	70 (6.70)		
CKM^l^ stage, n (%)				124.24 (3)^b^	<.001
0	985 (10.27)	945 (11.05)	40 (3.83)		
1	181(18.94)	1704 (19.93)	113 (10.82)		
2	3999 (41.69)	3488 (40.80)	511 (48.95)		
3	2792 (29.10)	2412 (28.21)	380 (36.40)		

a *t* test.

b Chi-square test.

c CRP: C-reactive protein.

d HbA_1c_: hemoglobin HbA_1c_.

e BUN: blood urea nitrogen.

f UA: uric acid.

g TC: total cholesterol.

h HDL: high-density lipoprotein.

i LDL: low-density lipoprotein.

j eGFR: estimated glomerular filtration rate.

k CVD: cardiovascular disease

l CKM: cardiovascular-kidney-metabolic.

The original model examined the relationship between depressive symptoms and the incidence of stroke. In this study, a total of 1044 participants were identified as having experienced a stroke. To evaluate the association between depressive scores and stroke incidence across CKM syndrome stages 0 to 3, 4 Cox proportional hazards models were constructed. Model I was adjusted for age, sex, marital status, educational level, and residential location. Model II expanded upon model I by incorporating additional variables, including smoking status and alcohol consumption. Model III further adjusted for laboratory parameters such as BUN, platelets, CRP, UA, HDL, LDL, triglycerides, and TC. Model IV extended model III by including comorbid chronic diseases such as hypertension, chronic liver disease, diabetes, and sleep problems. The study found a significant positive association between depression severity and the risk of stroke. When depression severity was treated as a continuous variable, the model yielded an HR of 1.03 (95% CI 1.02‐1.04; *P*<.001) in the fully adjusted model V.

Further clarifying the relationship between depressive score and stroke incidence, depressive score was categorized into quartiles. In a stratified analysis, the population was divided into 4 groups based on depression severity scores. The second group (scores 3‐7) showed no significant association with stroke incidence (HR 1.16, 95% CI 0.97‐1.40; *P*=.10), and this result remained unchanged after adjusting for covariates. In contrast, the third group (HR 1.54, 95% CI 1.29‐1.84; *P*<.001) and the fourth group (HR 1.69, 95% CI 1.41‐2.02; *P*<.001) both demonstrated significant associations, with the risk of stroke increasing as depression severity worsened. In the fully adjusted model V, compared to Q1, the HR for Q2, Q3, and Q4 were 1.13 (95% CI 0.94‐1.35), 1.49 (95% CI 1.24‐1.79), and 1.63 (95% CI 1.35‐1.96), respectively.

This indicates that participants in Q2, Q3, and Q4 experienced a 13%, 49%, and 63% higher risk of stroke compared to those in Q1 among the population with CKM syndrome stages 0 to 3. Except for the Q2 stage, the results for the other 2 stages were statistically significant (Table 3).

**Table 3.** Adjusted hazard ratios (HRs) for stroke incidence across depressive symptom quartiles in a population with cardiovascular-kidney-metabolic (CKM) syndrome stages 0 to 3.

Character	ScoreQ									
Stroke-years, depressive score	Crude model^a^		Model 1^b^		Model 2^c^		Model 3^d^		Model 4^e^	
	HR (95% CI)	*P* value	HR (95% CI)	*P* value	HR (95% CI)	*P* value	HR (95% CI)	*P* value	HR (95% CI)	*P* value
Q1	Reference		Reference		Reference		Reference		Reference	
Q2	1.16 (0.97-1.40)	.10	1.15 (0.96-1.38)	.13	1.15 (0.95-1.38)	.14	1.16 (0.97-1.40)	.11	1.13 (0.94-1.35)	.21
Q3	1.54 (1.29-1.84)	<.001	1.54 (1.28-1.84)	<.001	1.52 (1.27-1.83)	<.001	1.56 (1.30-1.87)	<.001	1.49 (1.24-1.79)	<.001
Q4	1.69 (1.42-2.02)	<.001	1.69 (1.41-2.02)	<.001	1.67 (1.39-2.00)	<.001	1.73 (1.45-2.08)	<.001	1.63 (1.35-1.96)	<.001

a Crude model: depressive score (Q)

b Model 1: depressive scoreQ, adjusted for age, sex, marital status, education, and location. *P* for trend (character2integer)<.001; *P* for trend (median value)<.001.

c Model 2: depressive scoreQ adjusted for age, sex, marital status, education, location, smoking status, and drinking status. *P* for trend (character2integer)<.001; *P* for trend (median value)<.001.

d Model 3: depressive scoreQ, adjusted for age, sex, marital status, education, location, smoking status, drinking status, blood urea nitrogen (BUN), C-reactive protein (CRP), uric acid (UA), total cholesterol (TC), triglycerides, low-density lipoprotein (LDL), and high-density lipoprotein (HDL). *P* for trend (character2integer)<.001; *P* for trend (median value)<.001.

e Model 4: depressive scoreQ, adjusted for age, sex, marital status, education, location, smoking status, drinking status, BUN, CRP, UA, TC, triglycerides, LDL, HDL, hypertension, sleep problem, liver disease, lung disease, and diabetes. *P* for trend (character2integer)<.001; *P* for trend (median value)<.001.

**Figure 2.**
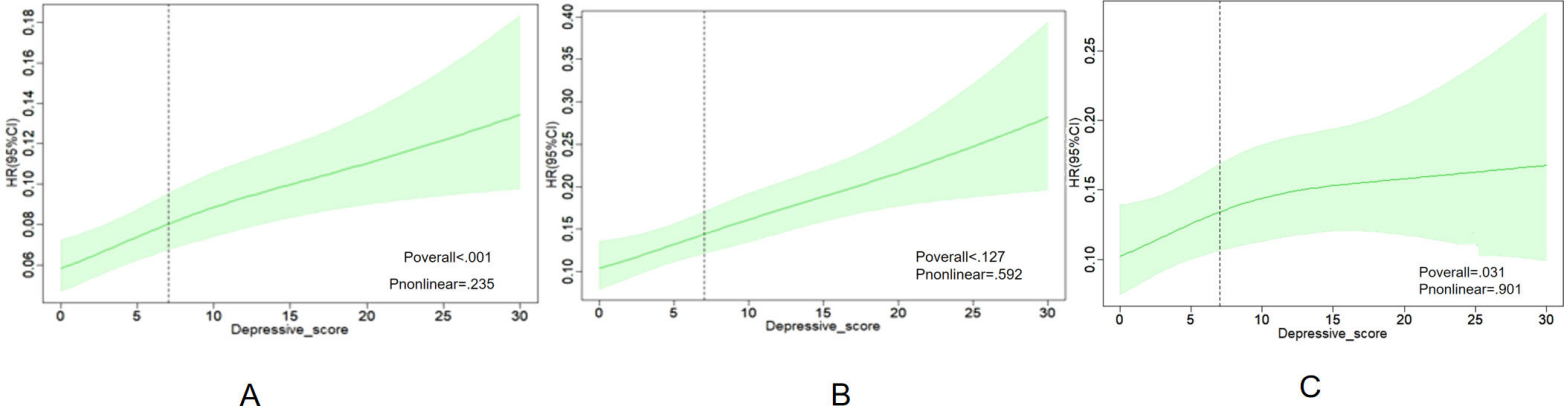
(A) The restricted cubic spline (RCS) analysis between the depressive score and stroke incidence in a population with cardiovascular-kidney-metabolic (CKM) syndrome stages 0‐3. The model was adjusted for age, sex, marital status, education, location, smoke, drink, blood urea nitrogen (BUN), C-reactive protein (CRP), uric acid (UA), total cholesterol (TC), triglycerides, low-density lipoprotein (LDL), high-density lipoprotein (HDL), hypertension, sleep problem, liver disease, lung disease, diabetes. (B) The RCS analysis between the depressive score and stroke incidence in a population with CKM syndrome stages 2. (C) The RCS analysis between the depressive score and stroke incidence in a population with CKM syndrome stages 3. HR: hazard ratio.

**Figure 3.**
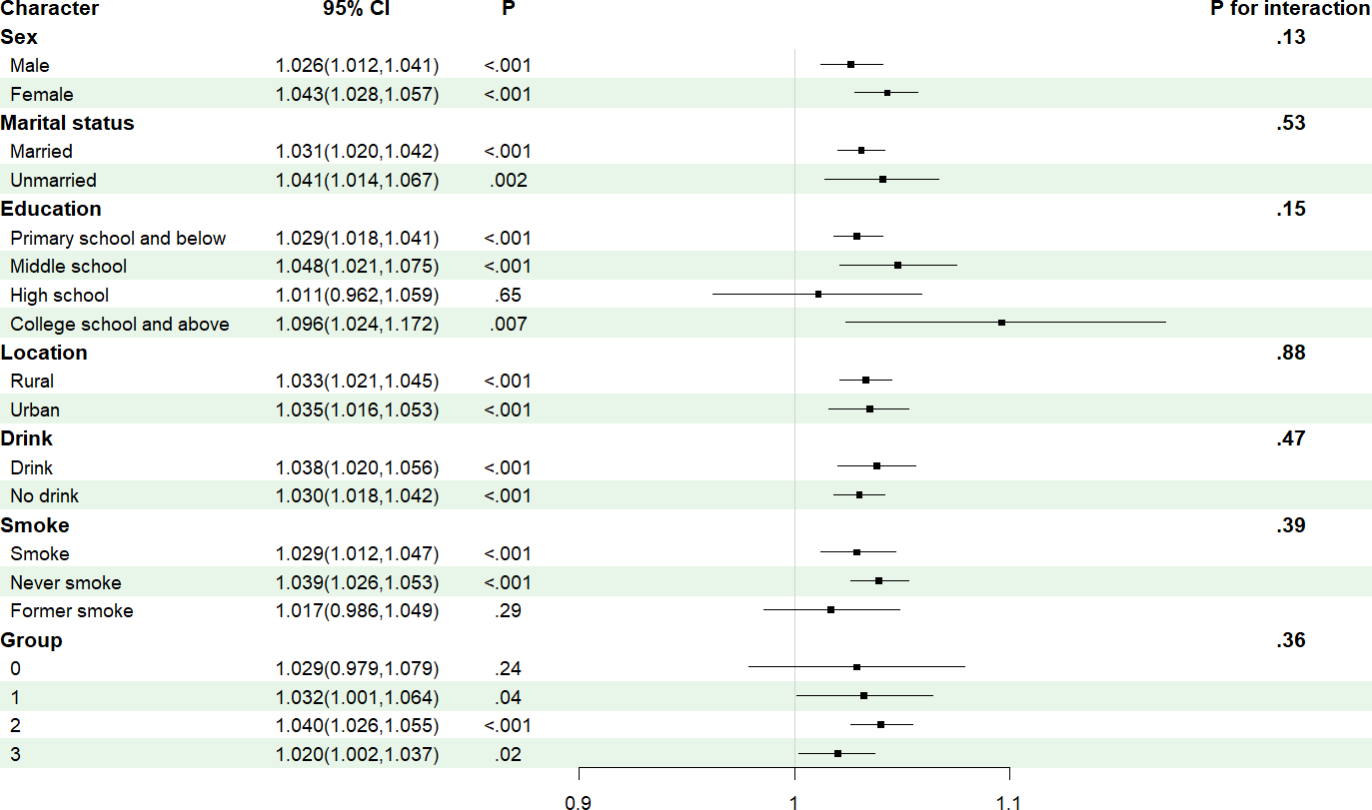
The forest plot of subgroup analyses of the association between the depressive score and stroke incidence in a population with cardiovascular-kidney-metabolic (CKM) syndrome stages 0 to 3.

RCS regression models were used to further examine the association between depressive score and the risk of stroke among participants with CKM syndrome. The fully adjusted RCS regression model revealed a significant positive linear association between depression scores and stroke risk across participants with CKM syndrome stages 0 to 3 (*P*_overall_<.001; *P*_nonlinear_=.20). Similarly, this positive linear correlation was evident in individuals at CKM syndrome stage 2 (*P*_overall_<.001*; P*_nonlinear_=.50) and stage 3 (*P*_overall_=.03; *P*_nonlinear_=.30; Figure 2). To further explore the relationship between and the incidence of stroke, subgroup and interaction analyses were conducted across different genders, smoking status, drinking status, chronic diseases, and CKM syndrome stages (stages 0-3) with no interactions observed in other subgroups (*P* for interaction >.05; Table 4 and Figure 3).

**Table 4.** Subgroup analyses of the association between the depressive score and stroke incidence in a population with cardiovascular-kidney-metabolic (CKM) syndrome stages 0 to 3.

Characteristic	HR (95% CI)	*P* value	*P* for interaction
Sex			.12
Male	1.026 (1.012-1.041)	<.001	
Female	1.043 (1.028-1.057)	<.001	
Marital status			.50
Married	1.031 (1.020-1.042)	<.001	
Unmarried	1.041 (1.014-1.067)	.002	
Education			.15
Primary school or below	1.029 (1.018-1.041)	<.001	
Middle school	1.048 (1.021-1.075)	<.001	
High school	1.011 (0.962-1.059)	.65	
College school and higher	1.096 (1.024-1.172)	.007	
Location			.88
Rural	1.033 (1.021-1.045)	<.001	
Urban	1.035 (1.016-1.053)	<.001	
Drinking status			.47
Drink	1.038 (1.020-1.056)	<.001	
No drink	1.030 (1.018-1.042)	<.001	
Smoke status			.39
Smoke	1.029 (1.012-1.047)	<.001	
Never smoke	1.039 (1.026-1.053)	<.001	
Former smoke	1.017 (0.986-1.049)	.29	
Stage			.36
0	1.029 (0.979-1.079)	.24	
3	1.020 (1.002-1.037)	.024	
2	1.040 (1.026-1.055)	<.001	
1	1.032 (1.001-1.064)	.04	

## Discussion

### Principal Findings

This study extends current knowledge by establishing for the first time a significant association between depressive symptoms and stroke incidence in individuals with CKM syndrome—a condition marked by the intricate interplay of cardiovascular, kidney, and metabolic dysfunctions. Through multivariable-adjusted Cox proportional hazards regression analyses controlling for demographic characteristics (age, sex, and education), lifestyle factors (smoking and physical activity), and clinical parameters (blood pressure, glycemic status, and renal function), we observed a 3% incremental stroke risk per 1-point increase in depressive symptom severity (adjusted HR 1.03, 95% CI 1.02‐1.04; *P*<.001). Notably, quartile-stratified analyses revealed a striking dose-response gradient. Participants in the highest depressive symptom quartile exhibited 63% greater stroke risk compared to the lowest quartile (HR 1.63, 95% CI 1.35‐1.96; *P*_trend_<.001), with intermediate quartiles demonstrating proportionally elevated risks (Q2: HR 1.13, 95% CI 0.94-1.35; Q3: HR 1.49, 95% CI 1.24-1.79). This monotonic risk escalation suggests a potential threshold effect where severe depressive symptoms may constitute a clinically meaningful cerebrovascular risk marker in populations with CKM.

This potential bidirectional relationship highlights the complex interplay between depression and stroke, suggesting that while depressive symptoms may precede stroke, the physiological and psychological impact of a stroke event may also exacerbate depressive states. In depression, disruptions in the serotonin system [28] and hyperactivation of the hypothalamic-pituitary-adrenal axis [29], characteristic of depression, may lead to elevated levels of inflammatory cells and proinflammatory cytokines such as IL-6 and IL-10 [30], both of which are closely associated with an increased risk of stroke. Chronic inflammation accelerates vascular injury and promotes atherosclerosis, significantly elevating the risk of both ischemic and hemorrhagic stroke. Additionally, excessive reactive oxygen species–induced oxidative stress further amplifies this risk by damaging endothelial cells and compromising vascular integrity [31]. Conversely, the inflammatory response following a stroke can deplete L-tryptophan, the precursor of serotonin [32], thereby worsening serotonin system dysfunction and contributing to depressive symptoms such as anhedonia [33]. Regarding the relationship between depression and kidney disease. A systematic review and meta-analysis found that in patients with CKD, depressive symptoms are significantly associated with higher levels of inflammatory markers, suggesting that inflammation may be a shared underlying mechanism linking depression with both CKD and stroke [34]. A study on patients with hypertension found that higher serum cortisol levels are associated with lower eGFR and poorer renal function [35]. Similarly, the pathological mechanisms of the CKM syndrome involve persistent chronic inflammation, oxidative stress, and endocrine dysfunction [36]. Abnormal fat accumulation in CKM serves as a major source of proinflammatory cytokines, exacerbating systemic inflammatory responses and further impairing cardiovascular and renal function [37]. However, this study did not observe a significant positive correlation between the severity of depressive symptoms and stroke incidence in patients with CKM stage 0. Chronic inflammation associated with CKM, along with substantial alterations in individual metabolic or cardiovascular risk factors, may play a pivotal role in mediating this relationship. Further research is warranted to elucidate these complex interactions and to identify potential intervention targets to mitigate stroke risk in this population.

Our analysis revealed that participants experiencing severe depressive symptoms in the CKM syndrome group had an increased risk of stroke compared to those with less severe symptoms. Stratified subgroup analyses (eg, age and gender) further confirmed that depressive symptoms significantly correlate with new-onset stroke incidence. These findings emphasize that depression is an independent risk factor for stroke, even when other systemic diseases are present. Addressing mild-to-moderate depressive symptoms early to prevent progression could significantly reduce the risk of stroke in populations with CKM syndrome, potentially mitigating both individual and systemic health burdens.

While numerous studies have documented a connection between depression and stroke, these investigations often overlook the broader systemic interactions underlying this relationship. Most previous studies have focused on the impact of poststroke depression on patient outcomes. Sun et al identified that disruptions in neurovascular coupling following a stroke may be a key mechanism underlying the development of depressive symptoms [38]. Approximately 27% of individuals who had a stroke experienced depression, with early-onset depression often persisting [39]. While research on the impact of depression on stroke incidence remains limited, most studies are cross-sectional. Persistently high depressive symptoms and remitted depressive symptoms were associated with increased stroke risk in a large cohort of US adults [40]. A meta-analysis encompassing 28 prospective cohort studies found that depression is associated with a 55% increase in the risk of stroke-related mortality [41]. Using the CHARLS database and logistic regression methods, Cui et al [42] identified a strong association between persistently high levels of depressive symptoms and increased stroke risk. However, the study overlooked the time-dependent relationship between depressive symptoms and stroke onset. In this study, we used a Cox proportional hazards model to examine the association between baseline depressive symptoms and the incidence of stroke, which accounts for time-dependent covariates and is better suited to complex clinical research scenarios.

This study offers several strengths. First, by using data from a large-scale prospective longitudinal study, we used Cox regression models to establish a positive association between depressive symptoms and stroke incidence. Notably, this is the first study to consider the influence of time-dependent variables on outcomes within a syndrome-specific population, thereby improving its clinical relevance. Second, we investigated both the overall relationship between depressive symptoms and stroke risk and the association across different quartiles of depressive symptom severity, allowing for a more nuanced understanding of how varying levels of depression impact stroke occurrence. Finally, subgroup analyses were performed to confirm the robustness and reliability of our findings.

This study has several limitations. First, previous research, including the China Kadoorie Biobank study [43] and other studies on Chinese populations, highlights the low proportion of individuals with depression who receive treatment [44]. As a result, the impact of antidepressant therapy on the study outcomes is likely minimal. Second, the diagnoses of stroke, diabetes, and hypertension in this study were based on self-reported information from consultations with medical professionals, which may introduce reporting bias. To address this limitation, future research should incorporate more objective diagnostic measures. Additionally, depressive symptoms were assessed using a single baseline Center for Epidemiologic Studies Depression Scale score, which may not capture dynamic longitudinal changes during the follow-up period. However, baseline measurements are validated as independent predictors in large-scale cohorts and represent a critical window for early clinical screening and risk stratification. Moreover, the incidence of stroke, as well as diagnoses of hypertension and diabetes, relied on self-reported data, which could lead to recall and misclassification biases. Finally, despite adjusting for a wide range of covariates, residual confounding may still exist due to unmeasured factors, such as specific dietary patterns, physical activity, and the use of medications like antidepressants or antihypertensives. While previous research suggests that antidepressant treatment rates are particularly low in Chinese populations, minimizing their potential impact on our findings, future studies should integrate more objective diagnostic measures and longitudinal data to further strengthen the robustness of these associations.

### Conclusions

This cohort study provides evidence of a significant association between depressive symptoms and an increased risk of stroke in individuals with CKM syndrome stages 0 to 3. The new AHA definition underscores the impact of emotional management on systemic, multisystem diseases. Effective treatment of depression may indirectly reduce the risk of stroke by lowering inflammation levels and promoting healthier lifestyle changes.

## Abbreviations

AHA: American Heart Association
CHARLS: China Health and Retirement Longitudinal Study
CKD: chronic kidney disease
CKM: cardiovascular-kidney-metabolic
CRP: C-reactive protein
CVD: cardiovascular disease
eGFR: estimated glomerular filtration rate
HbA_1c_: hemoglobin A_1c_
HDL: high-density lipoprotein
HR: hazard ratio
LDL: low-density lipoprotein
RCS: restricted cubic spline
TC: total cholesterol
UA: uric acid

## References

[1] Ndumele CE, Neeland IJ, Tuttle KR (2023). A synopsis of the evidence for the science and clinical management of cardiovascular-kidney-metabolic (CKM) syndrome: a scientific statement from the American Heart Association. Circulation.

[2] Nakashima A, Kato K, Ohkido I, Yokoo T (2021). Role and treatment of insulin resistance in patients with chronic kidney disease: a review. Nutrients.

[3] Lin L, Pan X, Feng Y, Yang J (2024). Chronic kidney disease combined with metabolic syndrome is a non-negligible risk factor. Ther Adv Endocrinol Metab.

[4] Pammer LM, Lamina C, Schultheiss UT (2021). Association of the metabolic syndrome with mortality and major adverse cardiac events: a large chronic kidney disease cohort. J Intern Med.

[5] Meikle PJ, Summers SA (2017). Sphingolipids and phospholipids in insulin resistance and related metabolic disorders. Nat Rev Endocrinol.

[6] Zewinger S, Schumann T, Fliser D, Speer T (2016). Innate immunity in CKD-associated vascular diseases. Nephrol Dial Transplant.

[7] Kadowaki T, Maegawa H, Watada H (2022). Interconnection between cardiovascular, renal and metabolic disorders: a narrative review with a focus on Japan. Diabetes Obes Metab.

[8] Cersosimo E, DeFronzo RA (2006). Insulin resistance and endothelial dysfunction: the road map to cardiovascular diseases. Diabetes Metab Res Rev.

[9] Malik S, Wong ND, Franklin SS (2004). Impact of the metabolic syndrome on mortality from coronary heart disease, cardiovascular disease, and all causes in United States adults. Circulation.

[10] Ostrominski JW, Arnold SV, Butler J (2023). Prevalence and overlap of cardiac, renal, and metabolic conditions in US adults, 1999-2020. JAMA Cardiol.

[11] Roth S, M’Pembele R, Matute P, Kotfis K, Larmann J, Lurati Buse G (2024). Cardiovascular-kidney-metabolic syndrome: Association with adverse events after major noncardiac surgery. Anesth Analg.

[12] Sarafidis P, Papadopoulos CE, Kamperidis V, Giannakoulas G, Doumas M (2021). Cardiovascular protection with sodium-glucose cotransporter-2 inhibitors and mineralocorticoid receptor antagonists in chronic kidney disease: a milestone achieved. Hypertension.

[13] Major RW, Cheng MRI, Grant RA (2018). Cardiovascular disease risk factors in chronic kidney disease: a systematic review and meta-analysis. PLoS ONE.

[14] Feigin VL, Brainin M, Norrving B (2022). World Stroke Organization (WSO): global stroke fact sheet 2022. Int J Stroke.

[15] Wu S, Wu B, Liu M (2019). Stroke in China: advances and challenges in epidemiology, prevention, and management. Lancet Neurol.

[16] Charlson FJ, Baxter AJ, Cheng HG, Shidhaye R, Whiteford HA (2016). The burden of mental, neurological, and substance use disorders in China and India: a systematic analysis of community representative epidemiological studies. Lancet.

[17] Flandreau EI, Ressler KJ, Owens MJ, Nemeroff CB (2012). Chronic overexpression of corticotropin-releasing factor from the central amygdala produces HPA axis hyperactivity and behavioral anxiety associated with gene-expression changes in the hippocampus and paraventricular nucleus of the hypothalamus. Psychoneuroendocrinology.

[18] Li H, Xia N (2020). The role of oxidative stress in cardiovascular disease caused by social isolation and loneliness. Redox Biol.

[19] Taraz M, Taraz S, Dashti-Khavidaki S (2015). Association between depression and inflammatory/anti-inflammatory cytokines in chronic kidney disease and end-stage renal disease patients: a review of literature. Hemodial Int.

[20] Hansson GK (2017). Inflammation and atherosclerosis: the end of a controversy. Circulation.

[21] Barreto DV, Barreto FC, Liabeuf S (2010). Plasma interleukin-6 is independently associated with mortality in both hemodialysis and pre-dialysis patients with chronic kidney disease. Kidney Int.

[22] Gilsanz P, Kubzansky LD, Tchetgen Tchetgen EJ (2017). Changes in depressive symptoms and subsequent risk of stroke in the cardiovascular health study. Stroke.

[23] Beekman AT, Deeg DJ, Van Limbeek J, Braam AW, De Vries MZ, Van Tilburg W (1997). Criterion validity of the Center for Epidemiologic Studies Depression scale (CES-D): results from a community-based sample of older subjects in The Netherlands. Psychol Med.

[24] Li W, Shen C, Kong W (2024). Association between the triglyceride glucose-body mass index and future cardiovascular disease risk in a population with cardiovascular-kidney-metabolic syndrome stage 0-3: a nationwide prospective cohort study. Cardiovasc Diabetol.

[25] Ma YC, Zuo L, Chen JH (2006). Modified glomerular filtration rate estimating equation for Chinese patients with chronic kidney disease. J Am Soc Nephrol.

[26] Luo H, Li J, Zhang Q (2018). Obesity and the onset of depressive symptoms among middle-aged and older adults in China: evidence from the CHARLS. BMC Public Health.

[27] Zhou L, Ma X, Wang W (2021). Relationship between cognitive performance and depressive symptoms in Chinese older adults: the China Health and Retirement Longitudinal Study (CHARLS). J Affect Disord.

[28] Ersoy B, Herzog ML, Pan W (2024). The atypical antidepressant tianeptine confers neuroprotection against oxygen-glucose deprivation. Eur Arch Psychiatry Clin Neurosci.

[29] Kim S, Park ES, Chen PR, Kim E (2022). Dysregulated hypothalamic–pituitary–adrenal axis is associated with increased inflammation and worse outcomes after ischemic stroke in diabetic mice. Front Immunol.

[30] Hassamal S (2023). Chronic stress, neuroinflammation, and depression: an overview of pathophysiological mechanisms and emerging anti-inflammatories. Front Psychiatry.

[31] Koutsaliaris IK, Moschonas IC, Pechlivani LM, Tsouka AN, Tselepis AD (2022). Inflammation, oxidative stress, vascular aging and atherosclerotic ischemic stroke. Curr Med Chem.

[32] Höglund E, Øverli Ø, Winberg S (2019). Tryptophan metabolic pathways and brain serotonergic activity: a comparative review. Front Endocrinol (Lausanne).

[33] Ko M, Choi-Kwon S, Jun SE (2018). Poststroke emotional disturbances and a tryptophan hydroxylase 2 gene polymorphism. Brain Behav.

[34] Jayakumar S, Jennings S, Halvorsrud K (2023). A systematic review and meta-analysis of the evidence on inflammation in depressive illness and symptoms in chronic and end-stage kidney disease. Psychol Med.

[35] Li X, Xiang X, Hu J (2016). Association between serum cortisol and chronic kidney disease in patients with essential hypertension. Kidney Blood Press Res.

[36] Sung CC, Hsu YC, Chen CC, Lin YF, Wu CC (2013). Oxidative stress and nucleic acid oxidation in patients with chronic kidney disease. Oxid Med Cell Longev.

[37] Spoto B, Di Betta E, Mattace-Raso F (2014). Pro- and anti-inflammatory cytokine gene expression in subcutaneous and visceral fat in severe obesity. Nutr Metab Cardiovasc Dis.

[38] Chao X, Fang Y, Lu Z (2024). Impairments of neurovascular coupling after stroke lower glymphatic system function and lead to depressive symptom: a longitudinal cohort study. J Affect Disord.

[39] Liu L, Xu M, Marshall IJ, Wolfe CD, Wang Y, O’Connell MD (2023). Prevalence and natural history of depression after stroke: a systematic review and meta-analysis of observational studies. PLoS Med.

[40] Gilsanz P, Walter S, Tchetgen Tchetgen EJ (2015). Changes in depressive symptoms and incidence of first stroke among middle‐aged and older US adults. J Am Heart Assoc.

[41] Pan A, Sun Q, Okereke OI, Rexrode KM, Hu FB (2011). Depression and risk of stroke morbidity and mortality: a meta-analysis and systematic review. JAMA.

[42] Cui Y, Zhu C, Lian Z (2021). Prospective association between depressive symptoms and stroke risk among middle-aged and older Chinese. BMC Psychiatry.

[43] Chen Y, Bennett D, Clarke R (2017). Patterns and correlates of major depression in Chinese adults: a cross-sectional study of 0.5 million men and women. Psychol Med.

[44] Phillips MR, Zhang J, Shi Q (2009). Prevalence, treatment, and associated disability of mental disorders in four provinces in China during 2001–05: an epidemiological survey. The Lancet.

